# Characterization of the complete chloroplast genome of sugar maple (*Acer saccharum*)

**DOI:** 10.1080/23802359.2019.1693932

**Published:** 2019-12-09

**Authors:** Xin Deng, Zhenxing Jiang, Jianchang Huang, Xianzhi Zhang

**Affiliations:** College of Horticulture and Landscape Architecture, Zhongkai University of Agriculture and Engineering, Guangzhou, China

**Keywords:** Chloroplast genome, *Acer saccharum*, phylogenomics

## Abstract

*Acer saccharum* is one ecologically and economically important tree species cultivated widely across the world. In this study we generated the complete chloroplast (cp) genome of *A. saccharum* via genome-skimming method. The assembled genome is 155,684 base-pairs (bp) in size, with one large single copy region of 85,393 bp and one small single copy region of 18,033 bp separated by two inverted repeats of 26,129 bp. The genome contains a total of 133 genes, including 85 protein-coding genes, 8 rRNAs and 40 tRNAs. Furthermore, phylogenomic estimation strongly supported *A. saccharum* as a distinct lineage within the monophyletic *Acer*.

## Main text

*Acer saccharum* Marshall, usually called sugar maple, is arguably one of the most ecologically and economically important tree species in the Northern Hemisphere (Wallace et al. 2018). It has also been widely cultivated as an ornamental plant across the world (Bishop et al. 2015). Despite its great value, to date the genetic and genomic data for this species are rather limited (Khodwekar et al. 2015). In the present study we generated the complete chloroplast (cp) genome of *A. saccharum* via genome-skimming method (Wang et al. 2018a), which will facilitate genetic studies and molecular breeding of sugar maple.

Fresh leaves of *A. saccharum* were collected from Kunming Botanic Garden in Kunming, Yunnan province of China (102°44′21″E, 25°08′15″N), for total genomic DNA extraction. The voucher specimen was preserved at the Tree Herbarium of Zhongkai University of Agriculture and Engineering (accession number ZXZ18018). Illumina paired-end (PE) library was constructed and sequenced by Beijing Genomics Institute (BGI) in Shenzhen, China. We assembled the cp genome using CLC Genomics Workbench v7.5 (CLC Bio, Aarhus, Denmark), as stated previously (Wang et al. 2018b). The assembled cp genome was then annotated via DOGMA (Wyman et al. 2004), coupled with manual correction.

The finished cp genome of *A. saccharum* (GenBank accession MN315280) is 155,684 base-pairs (bp) in size with high coverage (mean 380×), displaying a typical quadripartite organization: one large single copy region (LSC) of 85,393 bp and one small single copy region (SSC) of 18,033 bp separated by two inverted repeats (IRs) of 26,129 bp. The cp genome contains a total of 133 genes, with 85 protein-coding genes, 8 ribosomal RNA (rRNA) genes and 40 transfer RNA (tRNA) genes. Six protein-coding genes (*ndhB*, *rpl2*, *rpl23*, *rps7*, *ycf15*, *ycf2*) occur as duplicated genes in IRs and 12 ones are intron-containing genes (*atpF*, *clpP*, *ndhA*, *ndhB*, *petB*, *petD*, *rpl16*, *rpl2*, *rpoC1*, *rps12*, *rps16*, *ycf3*). The overall GC content of this cp genome is 37.9%. In short, the *A. saccharum* cp genome is structurally similar to previously published ones (Wang et al. 2017; Zhao et al. 2018).

**Figure 1.**
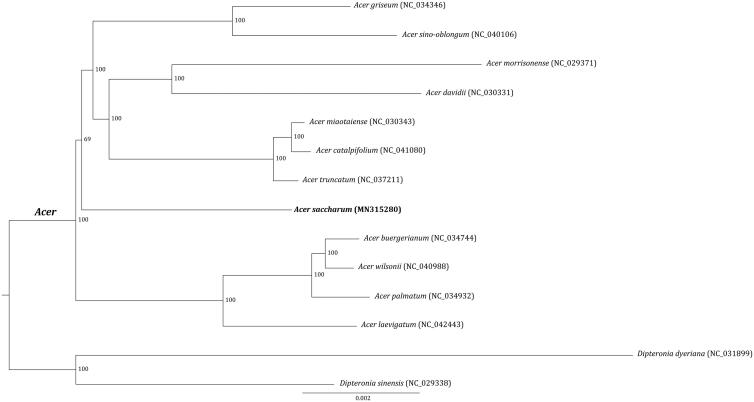
Maximum likelihood tree inferred from 14 Sapindaceae chloroplast genomes. The position of *Acer saccharum* is shown in bold. Values associated with nodes are bootstrapping support values.

Phylogenetic placement of *A. saccharum* was further estimated by RAxML v.8.2.8 (Stamatakis 2014) using 14 cp genomes of Sapindaceae. The resulted maximum likelihood (ML) tree supported that all the species of the genus *Acer* formed a monophyletic clade with high-support value (Figure 1). *A. saccharum* is a distinct lineage in *Acer*, being sister to a highly supported clade composed of eight species (*A. griseum*, *A. sino-oblongum, A. morrisonense*, *A. davidii*, *A. miaotaiense*, *A. catalpifolium*, *A. truncatum*, *A. deliciosa*). But it is worth noting that this relationship was just lowly supported (Figure 1). In all, the cp genome phylogenomics largely resolved the intractable phylogeny of *Acer*, consistent with previous studies (Zhao et al. 2018; Feng et al. 2019).
